# Enhanced biological performance of Sr^2+^-doped nanorods on titanium implants by surface thermal-chemical treatment

**DOI:** 10.1007/s10856-025-06898-z

**Published:** 2025-06-23

**Authors:** Xinrui Dai, Jianghui Zhao, Shengcai Qi, Ping Liu, Wei Li, Ke Zhang, Xiaohong Chen, Fengcang Ma

**Affiliations:** 1 School of Materials and Chemistry, University of Shanghai for Science and Technology, Shanghai, 200093 China; 2 Department of Prothodontics, Shanghai Stomatological Hospital, Fudan University, Shanghai, 200031 China; 3 Shanghai Key Laboratory of Craniomaxillofacial Development and Diseases, Fudan University, Shanghai, 200032 China

## Abstract

Titanium alloys, as artificial implants for orthopedic diseases, are prone to aseptic loosening and infection after surgery because their smooth surface restricts the attachment and movement of osteoblasts, resulting in a lack of osteogenic and antimicrobial properties. This study aimed to prepare SrTiO_3_ nanostructures with varying Sr content on the surface of titanium through a thermal-chemical treatment, enhancing the osteogenic capacity of titanium while providing antibacterial properties. The results indicated that the SrTiO_3_ nanostructures are primarily composed of pure titanium and SrTiO_3_ phases, exhibiting a rod-like surface morphology. Sr is uniformly distributed across the surface of the samples, and increasing the Sr content does not alter the morphology of the nanostructures. Wettability tests demonstrated that the SrTiO_3_ nanostructures exhibited superhydrophilicity, promoting cell adhesion. Electrochemical tests revealed that the SrTiO_3_ nanostructures prepared on the titanium surface significantly enhanced its corrosion resistance. After 14 days of immersion in simulated body fluids, a significant amount of hydroxyapatite formed on the surface of STN3, indicating that the SrTiO_3_ nanostructures possess good bioactivity. In vitro antimicrobial tests demonstrated that SrTiO_3_ nanostructures were effective against both *Escherichia coli* and *Staphylococcus aureus*, with the antimicrobial rates increasing alongside the Sr content, reaching 48.1% and 38.6%, respectively.

**Graphical Abstract:**

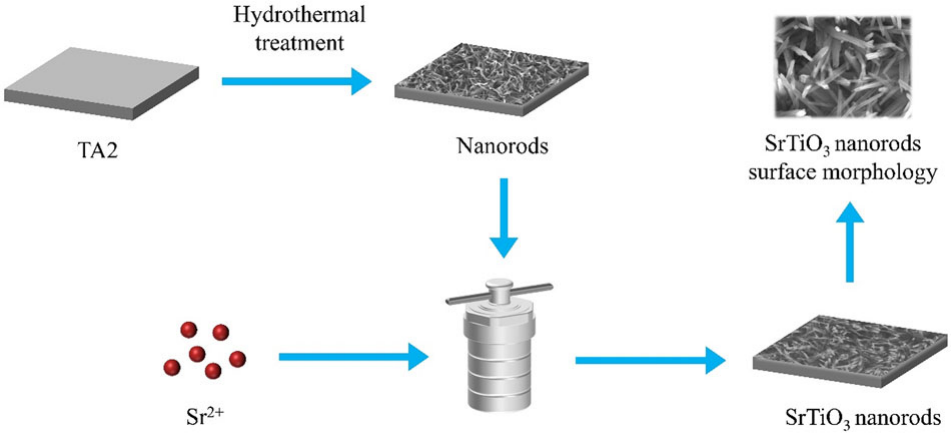

## Introduction

The aging population has led to an increase in orthopedic diseases such as osteoporosis, arthritis, and fractures, consequently raising the demand for bone implants [1]. Titanium alloys, characterized by low density, high specific strength, excellent corrosion resistance, and good biocompatibility, are extensively used as metal implants in orthopedic surgeries, including hip, knee, and spinal fixation devices [2, 3]. These alloys form a natural passivation film on their surface, which provides excellent corrosion resistance [4]. However, in the complex environment of the human body, body fluids can damage the natural passivation film on the surface of titanium alloys, leading to a reduction in corrosion resistance, and the resulting corrosion products can trigger inflammatory reactions and bone loss [5–7]. Severe inflammatory reactions can induce tissue hypoxia, creating a conducive environment for bacterial proliferation and indirectly increasing the risk of implant infection [8]. Bone loss can directly compromise the osseointegration between the bone and the implant, resulting in reduced implant stability and subsequent loosening [9, 10]. Therefore, it is essential to minimize the risk of infection in orthopedic implants, enhance their osseointegration performance, and reduce the postoperative failure rate. Surface modification can alter one or more properties, including surface morphology, wettability and corrosion resistance, which greatly affects the in vitro and in vivo performance of implants by adjusting the interaction with the surrounding environment and biological tissues [11]. The optimization of the film to enhance osseointegration, along with the introduction of antimicrobial surface treatments, not only improves implant stability but also effectively reduces the risk of postoperative infection [12].

A variety of biomedical surface modifications have been studied for titanium implants, with the surface engineering of titanium dioxide nanostructures showing significant potential [13, 14]. TiO_2_ nanotube structure with greater specific surface area promotes cell attachment and dissemination and has been shown to enhance osteoblast function [15]. TiO_2_ micro-pit and nano-nodular combined surface mimic the micro and nanoscale characteristics of natural bone tissue, creating a more favorable growth environment for osteoblasts and enhancing their functional activity, thereby promoting osteoblast proliferation [16, 17]. Indeed, the surface of TiO_2_ nanorod with well-arranged atomic stacks can induce protein adsorption, establish biosignalling channels for cellular osteogenesis and influence stem cell behavior [18]. Zhang et al. [19] prepared an entire array of TiO_2_/MoS_2_/PDA/RGD nanorods on titanium plates by hydrothermal treatment. The nanorods were irradiated under 660 nm visible light and 808 nm near infrared light, and the nanorods showed good antimicrobial activity both In vivo and In vitro. The nanorod arrays improved cell adhesion, proliferation and osteogenic differentiation by cellular experiments and enhanced osteogenic properties. Ge et al. [20] prepared TiO_2_ nanorod film by hydrothermal treatment and later doped mesoporous bioactive glass (MBG) into TiO_2_ nanorod film by sol-gel method, confirming that the surface morphology can be altered in this way to modulate cellular responses and improve osteoinductive capacity. Therefore, TiO_2_ nanorods have great potential to enhance the properties associated with bone implants.

In the human body, calcium is weakly regulated regarding bone resorption, yet it plays a direct role in the mineralization of the bone matrix. Similarly, strontium (Sr), like calcium (Ca), is an alkaline-earth metal classified in Group IIA of the periodic table [21]. As a result, it shares a similar chemical structure and polarity with calcium. Despite strontium is present in bone at about 0.01 per cent of bone mass, it is an essential trace element in human bone tissue [22, 23]. Sr reduces osteolysis and results in bone remodeling [24]. To a certain extent, Sr^2+^ can participate in the physiological activities of bone metabolism as a bone-inducing factor instead of Ca^2+^, promoting the differentiation of osteoblasts and mineralisation of bone matrix, and hindering the growth and differentiation of osteoclasts, and contributing more to the reduction of bone resorption [25, 26]. Therefore, Sr has attracted great clinical interest in stimulating bone formation.

In this research, Sr^2+^ doped nanostructures were prepared on the surface of pure titanium by hydrothermal treatment, and the surface structure, chemical composition, wettability, corrosion resistance and biological activity of the samples were tested and discussed. And then it was immersed in simulated body fluid (SBF) to evaluate the ability of bone-like apatite formation on the surface of the samples. Finaly, the antimicrobial capacity of SrTiO_3_ nanostructures was evaluated through in vitro antimicrobial experiments.

## Materials and methods

### Preparation TiO_2_ nanorods doped with Sr^2+^ on Ti substrate

Pure titanium sheets (TA2) were sectioned into dimensions of 10 mm × 10 mm × 3 mm. The surfaces of the samples were sequentially sanded using silicon carbide (SiC) sandpaper with grit sizes of 400 #, 800 #, 1000 #, 1500 #, and 2000 #. Subsequently, the samples were subjected to ultrasonic cleaning with anhydrous alcohol followed by deionized water. The treated titanium sheets were placed in a 25 mL reactor, to which 15 mL of 1 M NaOH solution was added. The reactor was then maintained at 220 °C for 4 h to form samples coated with Na_2_TiO_3_ nanostructures. Subsequently, the samples were immersed in 0.5 M HCl for 30 min to exchange Na^+^ with H^+^, resulting in samples covered with H_2_TiO_3_ nanostructures, which were designated as TN.

The samples covered with H_2_TiO_3_ nanostructures were placed in a 25 mL reactor and ion-exchanged by adding 15 mL of 0.01, 0.1 and 1 M (CH_3_COO)_2_Sr solution at 100 °C for 24 h. The samples doped with different concentrations of Sr^2+^ were obtained and named as STN1, STN2 and STN3. The nomenclature and hydrothermal treatment stage parameters for all samples are shown in Table 1.

**Table 1:** Stages of hydrothermal treatment performed and samples references

Sample	First step	Second step	Thrid step
TN	1 M 4 h/220 °C	0.5 M 30 min/25 °C	—
STN1	1 M 4 h/220 °C	0.5 M 30 min/25 °C	0.01 M 24 h/100 °C
STN2	1 M 4 h/220 °C	0.5 M 30 min/25 °C	0.1 M 24 h/100 °C
STN3	1 M 4 h/220 °C	0.5 M 30 min/25 °C	1 M 24 h/100 °C

### Morphology and structure characterization

The surface morphology of the samples was examined using scanning electron microscopy (SEM, Quanta FEG 450), while the elemental composition of the surface was quantitatively analyzed through energy-dispersive X-ray spectrometry (EDS, SDD Inca X-Max 50). The crystal structure of the samples was characterized by X-ray diffraction (XRD, D8 Advance) with a scanning range of 20–80 ° and a scanning speed of 5 °/min. Additionally, the films were investigated using X-ray photoelectron spectroscopy (XPS, EscaLab 250Xi), and the elemental composition and chemical states of the layers were determined using the C 1 s peak at 284.8 eV as the binding energy calibration reference.

### Wettability

At room temperature, 0.01 mL of simulated body fluid (SBF) was carefully dispensed onto the surface of each sample, and the wettability of the film was assessed using an interfacial tension tester (JC2000C1, Shanghai Metallic Environment Co., Ltd.). For each sample, measurements were conducted three times, with each measurement performed on a distinct sample.

### Sr^2+^ release

The samples were immersed in 20 mL of SBF for 1, 3, 7, 14, 21, and 28 days. The concentration of Sr^2+^ released into the SBF was measured using an inductively coupled plasma atomic emission spectrometer (ICP-AES, Perkin Elmer).

### Corrosion resistance analysis

Electrochemical testing of the samples was performed using an electrochemical workstation with a conventional three-electrode system (CHI600E, Shanghai Chenhua Instrument Co., Ltd.), and the corrosion resistance was evaluated based on electrochemical parameters derived from linear extrapolation of the Tafel curve. The saturated calomel electrode (SCE) was used as the reference electrode, a platinum electrode served as the counter electrode, and the sample under investigation acted as the working electrode. In the electrochemical test, the voltage scan rate was set to 10 mV/s within the open circuit potential (OCP) range of −1.0 V to 1.0 V. The sample had a working area of 1 cm², and the corrosion electrolyte used was simulated body fluid (SBF) at 37 °C. Prior to testing, the samples were immersed in SBF for 60 min. Each sample was tested three times, with a fresh sample used for each test to ensure experimental consistency and accuracy.

### Bioactivity test

The SBF was prepared according to the method described by Kokubo [27], and the in vitro bioactivity of the films in the four sample groups was evaluated through an SBF immersion test. In the immersion test, the samples were submerged in 5 mL of SBF at 37 °C for 1, 3, 7, and 14 days. After gold sputtering, the surfaces of the TN, STN1, STN2, and STN3 films were analyzed using scanning electron microscopy (SEM). Additionally, the films were examined by X-ray diffraction (XRD) and Fourier-transform infrared (FT-IR) spectroscopy to assess the formation of bone-like hydroxyapatite (HAp) ceramic films.

### In vitro antimicrobial activity test

In vitro antimicrobial assays were conducted using Gram-negative *Escherichia coli (E. coli)* and Gram-positive *Staphylococcus aureus (S. aureus)* to evaluate the antimicrobial efficacy of the film. After sterilization, the bacteria used in the experiments were inoculated into a liquid medium and incubated in a water-bath shaker at 37 °C and 90 r/min for 10 h. The bacterial concentration was then measured spectrophotometrically and diluted to 1 × 10^4^ CFU/mL. The samples were sterilized under ultraviolet light for 1 h, placed in 24-well plates with 2 mL of bacterial suspension, and incubated at 37 °C in a constant temperature incubator for 24 h. The bacteria were incubated on the sample surface for 24 h, then the bacterial suspension was aspirated, and the samples were rinsed with 2 mL of phosphate buffer saline (PBS) to detach the bacteria from the surface. The diluted bacterial solution was evenly spread onto the agar medium and incubated at 37 °C for 24 h to observe colony formation. The samples were then removed and fixed with 2.5% glutaraldehyde at 4 °C for 30 min, followed by bacterial dehydration using a graded series of ethanol solutions. After gold sputtering, the film of antibacterial mechanism was examined using SEM. The antimicrobial rate on the surface of the sample was calculated using the following formula:

1 $$R=(C0-{Cx})/{Cx}\times 100\, \%$$

In the formula, ${\rm{C}}0$ and ${\rm{Cx}}$ represent the number of colonies on nutrient agar plates for the control (TN) and the treated samples (STN), respectively.

### Statistical analysis

SPSS 25.0 software was used to analyze the single factor variance of the experimental data of each group. *P < 0.05 was considered statistically significant.

## Results and discussion

### Characterisation of film morphology

**Fig. 1:**
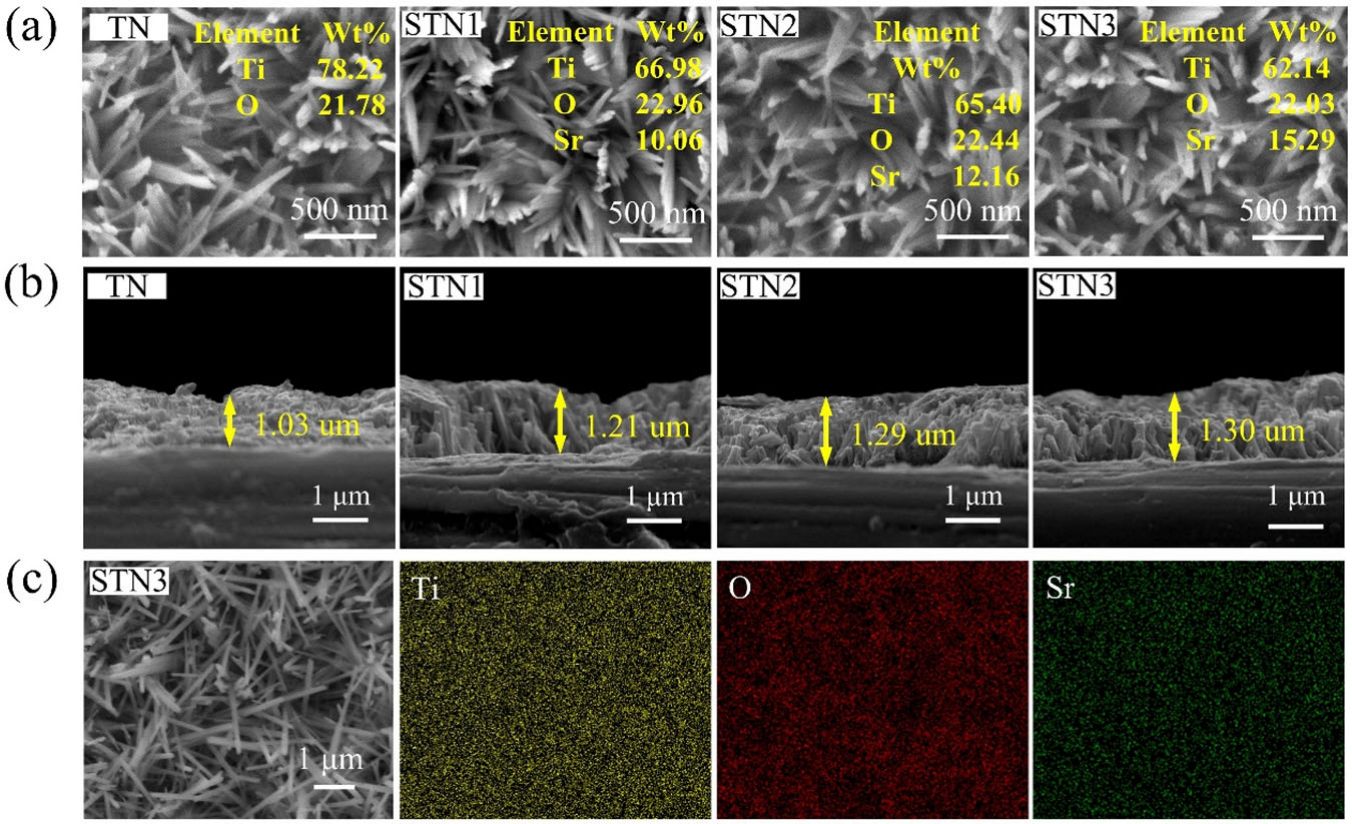
**a** Film surface of the four sample groups; (**b**) cross-section of the four sample groups; (**c**) SEM images and EDS mappings of STN3

The formation of nanostructures occurs in three processes, as described by Eqs. (2–4) [28, 29]. Figure 1a, b shows the nanorods doped with different concentrations of Sr^2+^, prepared via the hydrothermal treatment. All samples exhibit similar structural morphology, with the nanorods vertically distributed on the titanium sheet, forming a three-dimensional spatial arrangement. The nanorods on the TN surface had an average diameter of 51.24 ± 3.89 nm, while the diameters of the nanorods on the STN1, STN2, and STN3 surfaces were approximately 52.99 ± 2.88 nm. The cross-sectional size of the nanorods across all samples was around 1.21 ± 0.09 um. SEM results demonstrate that Sr^2+^ doping does not affect the morphology of the film. Analysis of the EDS energy spectra of the sample surface (Fig. 1c) revealed the presence of Ti and O on the surface of all four sample groups. In addition, Sr was detected in varying proportions in the STN samples. Ti, O, and Sr were uniformly distributed throughout the film. The results confirm that the hydrothermal treatment successfully doped Sr into the H_2_TiO_3_ nanostructures. Additionally, the Sr ion content in the film increased progressively with higher concentrations of (CH_3_COO)_2_Sr in the ion exchange solution. The content of Sr²⁺ in the film is not proportional to the concentration of the solution, owing to the combined influences of factors such as surface site availability and surface saturation [30, 31].

2 $${{\rm{Ti}}{\rm{O}}}_{2}+2{\rm{NaOH}}\to {{\rm{Na}}}_{2}{{\rm{TiO}}}_{3}+{{\rm{H}}2}{{\rm{O}}}$$

3 $${{\rm{Na}}}_{2}{{\rm{TiO}}}_{3}+2{\rm{HCl}}\to {{\rm{H}}}_{2}{{\rm{TiO}}}_{3}+2{\rm{NaCl}}$$

4 $${{\rm{H}}}_{2}{{\rm{TiO}}}_{3}+{({{\rm{CH}}}_{3}{\rm{COO}})}_{2}{\rm{Sr}}\to {{\rm{SrTiO}}}_{3}+{2{\rm{CH}}}_{3}{\rm{COOH}}$$

### Characterisation of the film structure

**Fig. 2:**
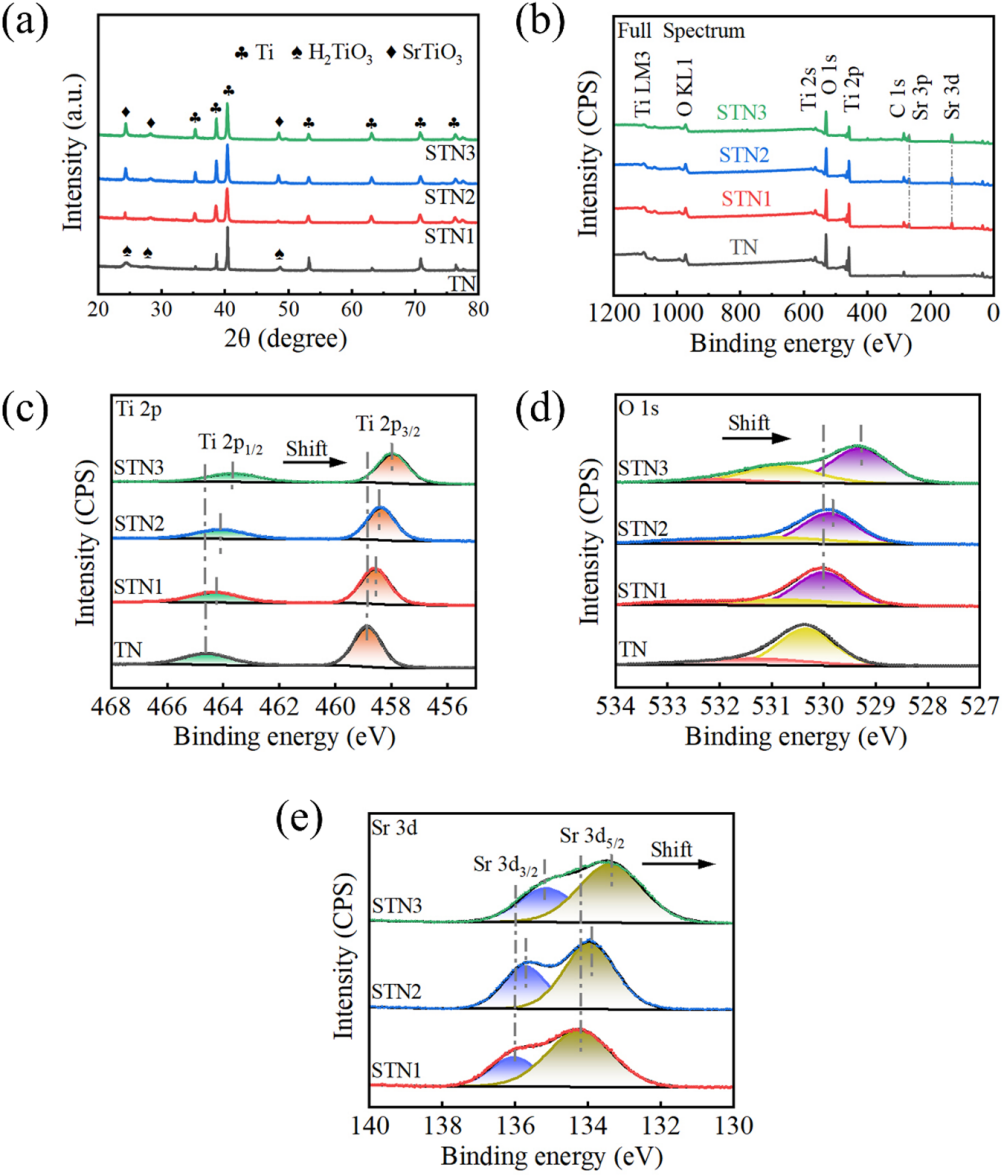
Four sample groups: (**a**) XRD patterns; (**b**) XPS full spectra; (**c**) Ti 2p; (**d**) O 1 s; (**e**) Sr 3 d

Figure 2a presents the XRD results of the four sample groups. TN consists of a pure titanium phase and a titanate phase, while STN1, STN2, and STN3 are composed of a pure titanium phase and a strontium titanate phase. This occurs as Sr^2+^ replaces H^+^ on the sample surface, forming SrTiO_3_ during the ion exchange process. Characteristic peaks of the SrTiO_3_ phase were observed at 24.4 °, 28.3 °, and 48.4 °. Among the three Sr^2+^-doped samples, STN3 exhibited the most intense SrTiO_3_ peaks, while STN1 showed the weakest peaks. This indicates that the concentration of Sr^2+^ influences the formation of strontium titanate. The elements and binding states in the films of the four sample groups were analyzed using XPS. The full and high-resolution spectra for all four groups are presented in Fig. 2b–e. The full spectra confirm the presence of Ti and O in all samples, with Sr also detected in the STN samples. The high-resolution spectra of the STN samples exhibit slight shifts compared to TN, which are attributed to the Sr^2+^ content in the film. With the increase of strontium ion doping concentration, the larger ionic radius of strontium ions leads to more and more significant changes in the local structure [32]. This change alters the electronic environment of strontium, and thus shifts the binding energy of the peak in XPS [33]. The presence of double peaks at 464.5 eV and 458.7 eV corresponding to Ti 2p_1/2_ and Ti 2p_3/2_ was observed in the high-resolution spectrum of Ti 2 P (Fig. 2c). In the O 1 s high-resolution spectra (Fig. 2d), the characteristic peak of TN at 530.3 eV corresponds to the Ti-OH bond, and the characteristic peak at 531.6 eV is induced by the adsorption of ambient H_2_O and O_2_ on the surface of the film. STN exhibits an additional characteristic peak compared to TN, which is attributed to the lattice oxygen (Sr-O-Ti) within the cubic structure of SrTiO_3_ [34]. The Sr 3 d spectra show two peaks at 133.4 eV and 135.2 eV (Fig. 2e). The prominent peak at 133.4 eV corresponds to the Sr 3d_5/2_ component of the Sr element. The shoulder peak at 135.2 eV corresponds to the Sr 3d_3/2_ component of the Sr element. The intensity of the main peak at 133.4 eV is significantly greater than that of the shoulder peak, indicating that Sr exists primarily as Sr^2+^ in the sample. This observation is consistent with the known chemical state of Sr in SrTiO_3_ [34, 35]. The intensity of the Sr 3 d characteristic peak directly reflects the Sr content in the sample. An increase in Sr^2+^ content leads to a higher peak intensity, indicating a greater presence of SrTiO_3_ in the film.

### Sr^2+^ release and wettability

**Fig. 3:**
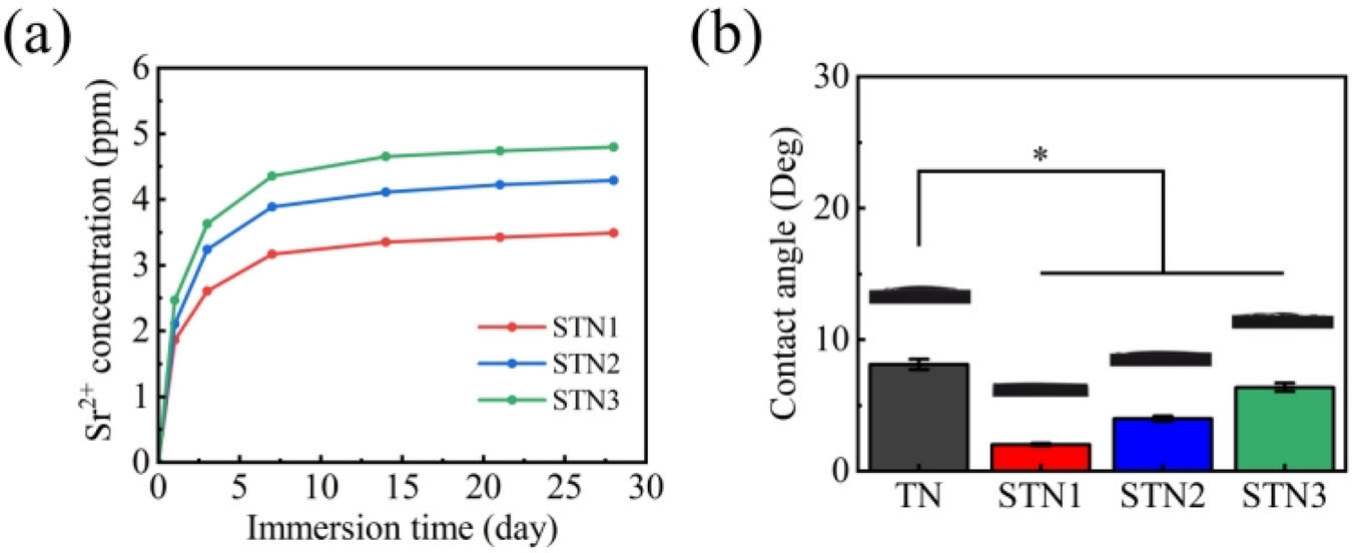
Different samples: (**a**) Sr^2+^ release curve in SBF; (**b**) water contact angles. The results are expressed as the means ± SDs (compared with the TN group, * *p* < 0.05)

Titanium and titanium alloys are commonly used as implants in orthopedic surgery. The release of metal ions post-surgery can inhibit bacterial growth and stimulate bone regeneration to some extent. However, this ion release may also cause allergic reactions, potentially posing risks to the patient. Therefore, studying the release of metal ions from these implants is crucial. The hydrolysis of SrTiO_3_ is comparable to that of other titanates, and the ion release process can be represented by Eq. (5) [36]. Figure 3a illustrates the concentration of Sr^2+^ released into SBF. The results indicate a significant increase in Sr^2+^ concentration over the first 14 days. Subsequently, the rate of increase slowed, suggesting that the release of Sr^2+^ from the film gradually slows over time, allowing for prolonged release into SBF. The slower release of Sr^2+^ may be attributed to the diminishing concentration gradient. Initially, the film has a higher concentration of Sr^2+^ compared to the SBF, but as the concentration difference decreases over time, the rate of Sr^2+^ release also slows down [37]. As immersion time increased, Sr^2+^ was progressively released from the film into SBF. Consequently, the concentration gradient between the film and SBF decreased over time, leading to a reduction in the ion release rate until it eventually stopped. At 28 days, the highest concentration of Sr^2+^ released from STN3 into SBF reached 4.79 ppm, which is in line with the safe concentration range for BMSCs in the reference [25]. In addition, literature have reported that Sr²⁺-doped coatings fabricated on titanium alloy surfaces demonstrated a cumulative Sr²⁺ release concentration of 14 ppm in SBF by day 9. Compared to undoped titanium alloys, rBMSCS cultured on the Sr²⁺-doped substrates exhibited significantly higher cell viability by day 7 [38].

5 $${{\rm{SrTiO}}}_{3}+{{\rm{H}}}_{2}{\rm{O}}\to {{\rm{Sr}}}^{2+}+{{\rm{TiO}}}_{2}+{2{\rm{OH}}}^{-}$$

The wettability of the samples was assessed using SBF, as shown in Fig. 3b. The results indicated that the water contact angle of TN was 8.12 °, demonstrating superhydrophilicity. This behavior is attributed to the high density of hydrophilic hydroxyl (-OH) groups on the surface of TN, which readily interact with water molecules through hydrogen bonding. This interaction facilitates the spreading of water molecules across the film, thereby reducing the water contact angle and exhibiting superhydrophilicity [39]. In addition, the dripping SBF will fill the gaps between the nanorods, further enhancing the wettability of the film. The water contact angle of the film decreases after doping with Sr^2+^ and increases with increasing Sr^2+^ concentration. However, the film remains superhydrophilic. This modest change may be attributed to the fact that at lower Sr^2+^ concentrations, the ions diffuse into the H_2_TiO_3_ lattices, creating lattice defects. These defects modify the surface energy of the film, resulting in a higher surface energy and consequently enhancing the wettability of the film [40]. At higher concentrations of Sr^2+^, the surface of the film exhibits a reduction in hydroxyl and oxygen vacancies. Consequently, the wettability decreases and the water contact angle increases [41].

### Corrosion resistance

**Fig. 4:**
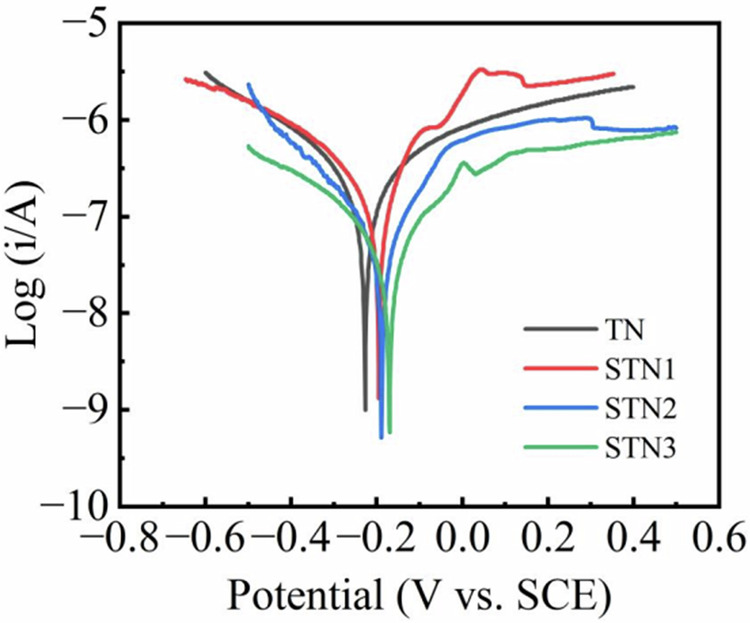
Polarization curves for TN, STN1, STN2 and STN3

The kinetic potential polarisation curves of the samples in SBF corrosive medium are shown in Fig. 4, and the electrochemical parameters fitted according to the linear extrapolation of the Tafel curves are shown in Table 2. Compared to TN, the corrosion current densities (I_corr_) for STN1, STN2, and STN3 were significantly reduced, with all values being lower than the I_corr_ of TN, which was 6.679 × 10⁻^8^ A/cm^2^. Concurrently, the self-corrosion potentials of STN1, STN2, and STN3 shifted positively. In general, corrosion potential and corrosion current density are key indicators for assessing a sample’s corrosion resistance [42, 43]. A higher positive corrosion potential and a lower corrosion current density suggest that the sample exhibits superior corrosion resistance. The polarisation curves were tested with a minimum corrosion current density (I_corr_) of 1.836 × 10⁻^8^ A/cm^2^ and a maximum self-corrosion potential (E_corr_) of −0.171 V for STN3. The test results demonstrated that doping with Sr^2+^ enhanced the corrosion resistance of the samples. Additionally, the corrosion potential increased progressively with higher Sr^2+^ concentrations. Among all the samples, STN3 exhibited the highest corrosion potential (E_corr_), indicating superior corrosion resistance in SBF media. This improvement is attributed to the formation of a stable passivation film on the nanorod surface by Sr^2+^, which limits the contact between the corrosive medium and the material, thereby enhancing corrosion resistance [44]. As the concentration of Sr^2+^ increases, this passivation film may become more intact and stable, thus further enhancing corrosion resistance.

**Table 2:** Electrochemical parameters of four groups of sample films (Potential vs. SCE)

	TN	STN1	STN2	STN3	ANOVA
EOCP(V)	−0.145	−0.140	−0.139	−0.112	* *p* < 0.05
E_corr_(V)	−0.227	−0.195	−0.191	−0.171	
I_corr_(A/cm^2^) ×10^−8^	6.679	6.456	2.566	1.836	

### In vitro mineralisation

**Fig. 5:**
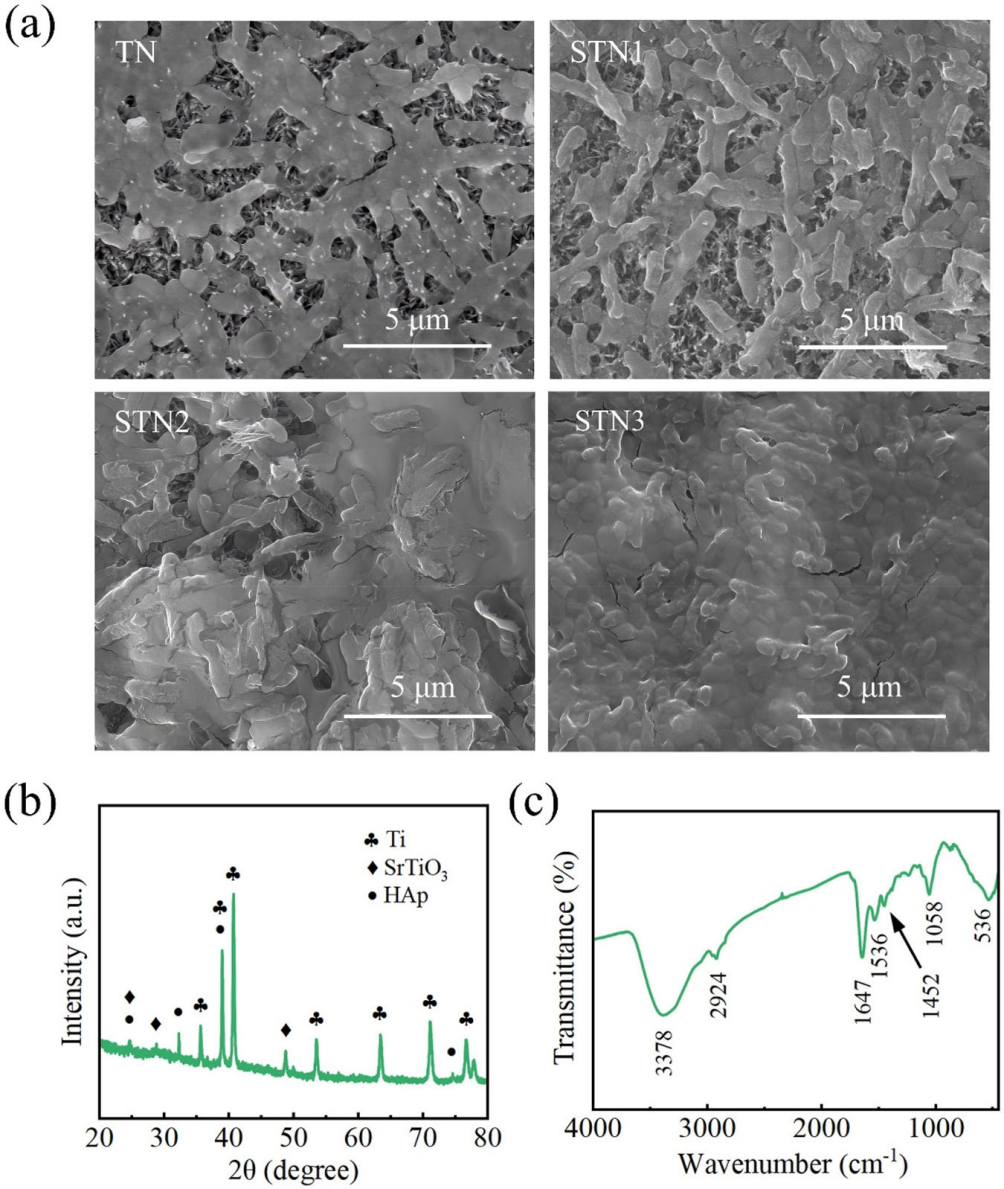
After immersion for 14 days: (**a**) surface morphology of TN, STN1, STN2 and STN3; (**b**) XRD pattern of STN3; (**c**) FT-IR spectrum of STN3

Figure 5a presents SEM images of TN, STN1, STN2, and STN3 after 14 days of immersion in SBF at 37 °C, revealing the formation of bone-like HAp deposits on the sample surfaces. Figure 5b shows the XRD patterns of the samples after 14 days of immersion period. Characteristic peaks of HAp were detected at 25.6 °, 31.8 °, and 53 °, corresponding to the (002), (211), and (004) crystal planes, respectively (JCPDS card no: 09–0432). This confirms that Sr^2+^ can induce the deposition of bone-like HAp, indicating that both TN and STN possess bioactivity [45]. The amount of bone-like HAp deposited on the surface of Sr^2+^-doped samples is greater than that on TN. This is attributed to Sr^2+^ having a similar ionic radius and chemical properties to Ca^2+^, allowing it to partially replace Ca^2+^ in the HAp lattice [46]. Moreover, this substitution has minimal impact on the crystal structure of HAp, while promoting crystal nucleation and growth during in vitro mineralization. The ability of these nanorods to induce bone-like HAp follows the order of STN3 > STN2 > STN1 > TN due to doping with different levels of Sr^2+^.

The FT-IR spectral detection result of the immersed STN3 film is shown in Fig. 5c. The characteristic absorption peaks of OH^-^ and PO_4_^3-^ are observed at 3378 cm^−1^ and 1058 cm^−1^, respectively, corresponding to the O-H bonding in OH^-^ and the stretching vibrations of the P-O bonds in PO_4_^3-^ [47, 48]. These results confirm that the surface of the STN3 is covered with a layer of HAp. In addition, the absorption peak observed near 2924 cm^−1^ corresponds to C-H stretching vibrations [49]. Due to the high levels of H_2_O and CO_2_ in the surrounding environment, water molecules are readily adsorbed onto the surface of the film [50, 51]. Consequently, the absorption peaks observed at 1536 cm^−1^ and 1647 cm^−1^ correspond to the bending vibrations of the hydroxyl groups in the adsorbed water molecules. Ambient CO_2_ dissolves into the surface of the film, resulting in the appearance of a characteristic absorption peak at 1452 cm^−1^, which corresponds to CO_3_^2-^. Bending vibrations associated with Ti-O bonding typically occur in the range of 400–500 cm^−1^ [49]. However, the doping of Sr^2+^ induces local lattice distortions, which may shift the absorption peak to 536 cm^−1^, possibly corresponding to the bending vibrations of Sr-O-Ti.

### In vitro antimicrobial properties

**Fig. 6:**
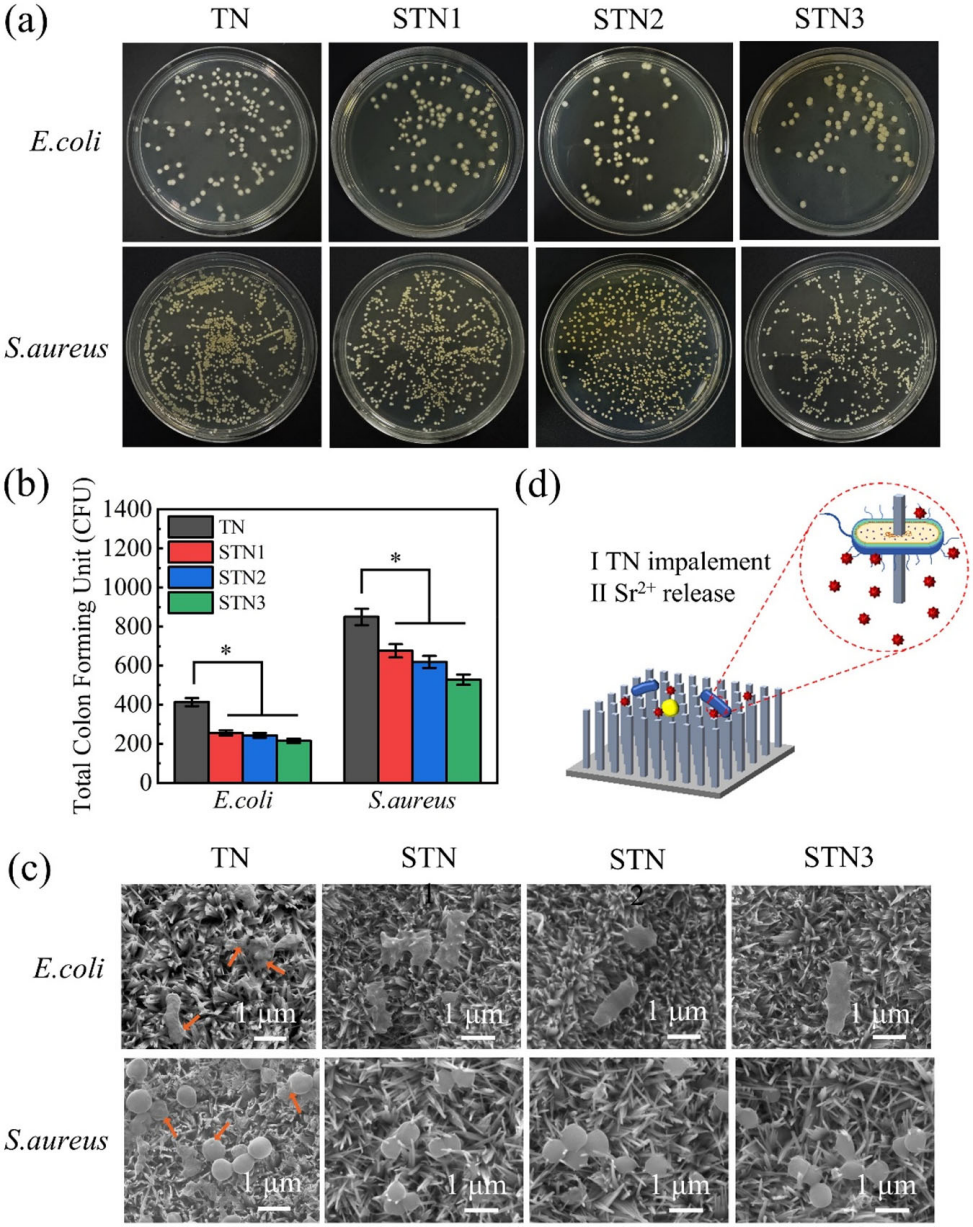
Four sample groups: (**a**) Antibacterial plate diagrams for *E. coli* and *S. aureus*; (**b**) number of colonies on the plate; (**c**) morphology of bacteria on the surface of the film; (**d**) diagram of antibacterial mechanism. The results are expressed as the means ± SDs (compared with the TN group, * *p* < 0.05)

Figure 6a, b shows the antimicrobial effect plate plots and colony counts of the four groups of samples against *E. coli* and *S. aureus*. The results indicated a gradual decrease in the number of colonies on the agar plates, demonstrating that doping with Sr^2+^ enhanced the antimicrobial capacity of the samples. The antimicrobial capacity of STN was superior to that of TN, with STN3 demonstrating a 48.1% inhibition of *E. coli* and a 38.6% inhibition of *S. aureus* compared to the TN control. The bacterial morphology on the membrane layer is shown in Fig. 6c, where SEM images clearly reveal the presence of intact *E. coli* and *S. aureus* on the membrane surface. *E. coli* appears in a rod-like shape, while *S. aureus* exhibits a spherical morphology. In the SEM image of the TN sample, a noticeable change in bacterial morphology is observed, with the bacteria shrinking from their originally full form, as indicated by the arrows. The SEM images revealed a marked morphological transformation in the TN sample, with bacterial cells at the arrow-marked regions transitioning from a plump to a shrunken appearance. The antibacterial mechanism of SrTiO_3_ nanorods, as shown in Fig. 6d, involves several factors that contribute to bacterial apoptosis. On one hand, the nanorod structure on the surface of the sample plays a role in physical antibacterial activity [52]. Initially, the nanorods are vertically arranged on the surface of the sample, with sharp tips. Upon contact with the bacteria, the cell membrane interacts with the sharp tips of the nanorods under its own weight. Over time, the contact area between the cell membrane and the nanorods increases, eventually leading to the disruption of the membrane structure as the nanorods penetrate and cause physical damage, ultimately resulting in bacterial cell death. On the other hand, the release of Sr²⁺ from the surface of the sample may also influence its antibacterial performance. As an alkaline earth metal ion, Sr²⁺ shares similar charge and ionic radius with Ca²⁺, and it may interfere with the calcium-dependent metabolic pathways in bacteria through a competitive mechanism [53]. Furthermore, the release of Sr²⁺ ions from the membrane, which carries a positive charge, can be easily adsorbed onto the negatively charged bacterial cell membrane due to electrostatic interactions between the positive and negative charges [54, 55]. This adsorption disrupts the charge balance and alters the membrane’s permeability [56]. Such changes can lead to cytoplasmic leakage, ultimately resulting in cell apoptosis.

## Conclusion

In this research, H_2_TiO_3_ nanorods were successfully synthesized on TA2 titanium alloy via a hydrothermal treatment process. Subsequently, SrTiO_3_ nanorods were prepared by treating the material with aqueous solutions of (CH_3_COO)_2_Sr at different concentrations. The surface morphology, structure, properties, as well as osteogenic and antibacterial performance of the synthesized nanorods were thoroughly characterized. The results show that the H_2_TiO_3_ nanorods mainly consist of a pure titanium and a titanate phase, while the Sr^2+^ doping did not alter their morphology. The SrTiO_3_ nanorods, in turn, are composed of both pure titanium and strontium titanate phases. Compared to H_2_TiO_3_ nanorods, SrTiO_3_ nanorods exhibited superior wettability and improved corrosion resistance in SBF. Both types of nanorods facilitated the deposition of bone-like HAp in these fluids, with the SrTiO_3_ nanorods (particularly the STN3 variant) inducing a higher amount of bone-like HAp formation. Furthermore, in vitro antimicrobial assays revealed that SrTiO_3_ nanorods effectively inhibited the growth of both *E. coli* and *S. aureus*, with STN3 exhibiting superior bacteriostatic efficacy effect. In conclusion, Sr^2+^-doped nanorods not only protect titanium alloys from severe corrosion but also enhance their bacteriostatic properties. These improvements significantly contribute to the bioinertness and overall performance of titanium alloys, making them more suitable as implant materials. This research highlights the promising potential of SrTiO_3_ nanorods in improving the osseointegration and clinical effectiveness of titanium-based implants.

## Data Availability

Data will be made available on request.

## References

[1] Cui YW, Wang LQ, Zhang LC. Towards load-bearing biomedical titanium-based alloys: From essential requirements to future developments. Prog Mater Sci. 2024;144:101277 10.1016/j.pmatsci.2024.101277

[2] Han X, Ma J, Tian A, Wang Y, Li Y, Dong B, et al. Surface modification techniques of titanium and titanium alloys for biomedical orthopaedics applications: A review. Colloids Surf B: Biointerfaces. 2023;227:113339 10.1016/j.colsurfb.2023.113339 37182380 10.1016/j.colsurfb.2023.113339

[3] Li K, Tao B, Tian H, Wu J, Huang K, Yan C, et al. Titanium implants with antiaging effect to repair senile osteoporosis fracture. Mater Des. 2023;232:112071 10.1016/j.matdes.2023.112071

[4] Li Y, Zhou Z, He Y. Tribocorrosion and surface protection technology of titanium alloys: a review. Materials. 2024;17:65 10.3390/ma17010065 10.3390/ma17010065 PMC10779822 38203919

[5] Du X, Shi W, Xiang S. Effect of low dissolved oxygen concentration on the defects and composition of regenerated passive film of Ti-6Al-4V alloy under continuous wear. RSC Adv. 2023;13:20135–49. 10.1039/D3RA03865C 37416911 10.1039/d3ra03865c PMC10320359

[6] Cvijovic´-Alagic´ I, Cvijovic´ Z, Bajat J, Rakin M. Composition and processing effects on the electrochemical characteristics of biomedical titanium alloys. Corros Sci. 2014;83:245–54. 10.1016/j.corsci.2014.02.017

[7] Liu X, Chu PK, Ding C. Surface modification of titanium, titanium alloys, and related materials for biomedical applications. Mater Sci Eng R Rep. 2004;47:49–121. 10.1016/j.mser.2004.11.001

[8] Hodges NA, Sussman EM, Stegemann JP. Aseptic and septic prosthetic joint loosening: Impact of biomaterial wear on immune cell function, inflammation, and infection. Biomaterials. 2021;278:121127 10.1016/j.biomaterials.2021.121127 34564034 10.1016/j.biomaterials.2021.121127

[9] Zemtsova EG, Kozlova LA, Yudintceva NM, Sokolova DN, Arbenin AY, Ponomareva AN, et al. Creation of a composite bioactive coating with antibacterial effect promising for bone implantation. Molecules. 2023;28:1416 10.3390/molecules28031416 36771083 10.3390/molecules28031416 PMC9919298

[10] Xiong S, Lu X, Zhang S, Cui Y, Chen J, Wei C, et al. B. Yang, Osteogenic properties of bioactive titanium in inflammatory environment. Dent Mater. 2023;39:929–37. 10.1016/j.dental.2023.08.180 37640634 10.1016/j.dental.2023.08.180

[11] Xu J, Zhang J, Shi Y, Tang J, Huang D, Yan M, et al. Surface modification of biomedical Ti and Ti alloys: A review on current advances. Materials. 2022;15:1749 10.3390/ma15051749 35268983 10.3390/ma15051749 PMC8911755

[12] Clainche TL, Linklater D, Wong S, Le P, Juodkazis S, Guével XL, et al. Mechano-bactericidal titanium surfaces for bone tissue engineering. ACS Appl Mater Interfaces 2020;12:48272–48283. 10.1021/acsami.0c11502 33054152 10.1021/acsami.0c11502

[13] Bachvarova-Nedelcheva A, Iordanova R, Naydenov A, Stoyanova A, Georgieva N, Nemska V, et al. Sol-Gel obtaining of TiO_2_/TeO_2_ nanopowders with biocidal and environmental applications. Catalysts. 2023;13:257 10.3390/catal13020257

[14] Xiao F, Ye J-H, Huang C-X, Dai J-H, Chen K-J, Xu X, et al. Gradient gyroid Ti6Al4V scaffolds with TiO_2_ surface modification: promising approach for large bone defect repair. Biomater Adv. 2024;161:213899 10.1016/j.bioadv.2024.213899 38772133 10.1016/j.bioadv.2024.213899

[15] Tao B, Lan H, Zhou X, Lin C, Qin X, Wu M, et al. Regulation of TiO_2_ nanotubes on titanium implants to orchestrate osteo/angio-genesis and osteo-immunomodulation for boosted osseointegration. Mater Des. 2023;233:112268 10.1016/j.matdes.2023.112268

[16] Kubo K, Tsukimura N, Iwasa F, Ueno T, Saruwatari L, Aita H, et al. Cellular behavior on TiO_2_ nanonodular structures in a micro-to-nanoscale hierarchy model. Biomaterials. 2009;30:5319–29. 10.1016/j.biomaterials.2009.06.021 19589591 10.1016/j.biomaterials.2009.06.021

[17] Iwasa F, Tsukimura N, Sugita Y, Kanuru RK, Kubo K, Hasnain H, et al. TiO_2_ micro-nano-hybrid surface to alleviate biological aging of UV-photofunctionalized titanium. Int J Nanomed. 2011;6:1327–41. 10.2147/IJN.S22099 10.2147/IJN.S22099 PMC3133524 21760728

[18] Cheng K, Yu M, Liu Y, Ge F, Lin J, Weng W, et al. Influence of integration of TiO_2_ nanorods into its nanodot films on pre-osteoblast cell responses. Colloids Surf B: Biointerfaces. 2015;126:387–93. 10.1016/j.colsurfb.2014.12.002 25511438 10.1016/j.colsurfb.2014.12.002

[19] Zhang G, Zhang X, Yang Y, Chi R, Shi J, Hang R, et al. Dual light-induced in situ antibacterial activities of biocompatibleTiO_2_/MoS_2_/PDA/RGD nanorod arrays on titanium. Biomater Sci. 2020;8:391–404. 10.1039/C9BM01507H 31728464 10.1039/c9bm01507h

[20] Ge F, Yu M, Yu C, Lin J, Weng W, Cheng K, et al. Improved rhBMP-2 function on MBG incorporated TiO_2_ nanorod films. Colloids Surf B: Biointerfaces. 2017;150:153–8. 10.1016/j.colsurfb.2016.11.030 27914251 10.1016/j.colsurfb.2016.11.030

[21] No YJ, Roohaniesfahani S, Lu Z, Shi J, Zreiqat H. Strontium-doped calcium silicate bioceramic with enhanced in vitro osteogenic properties. Biomed Mater. 2017;12:035003 10.1088/1748-605X/aa6987 28348275 10.1088/1748-605X/aa6987

[22] Sahoo S, Sinha A, Das M. Synthesis, characterization and in vitro biocompatibility study of strontium titanate ceramic: A potential biomaterial. J Mech Behav Biomed Mater. 2020;102:103494 10.1016/j.jmbbm.2019.103494 31654991 10.1016/j.jmbbm.2019.103494

[23] Stipniece L, Wilson S, Curran JM, Chen R, Salma-Ancane K, Sharma PK, et al. Strontium substituted hydroxyapatite promotes direct primary human osteoblast maturation. Ceram Int. 2021;47:3368–79. 10.1016/j.ceramint.2020.09.182

[24] Zhang W, Shen Y, Pan H, Lin K, Liu X, Darvell BW, et al. Effects of strontium in modified biomaterials. Acta Biomater. 2011;7:800–8. 10.1016/j.actbio.2010.08.031 20826233 10.1016/j.actbio.2010.08.031

[25] Yang X, Wang Q, Yan C, Huang D, Zhang Y, He H, et al. A dual-functional strontium-decorated titanium implants that guides the immune response for osseointegration of osteoporotic rats. Colloids Surf B: Biointerfaces. 2024;233:113643 10.1016/j.colsurfb.2023.113643 37995629 10.1016/j.colsurfb.2023.113643

[26] Liu Z, Ding H, Qi L, Wang J, Li Y, Liu L, et al. Core-shell NiS@SrTiO_3_ nanorods on titanium for enhanced osseointegration via programmed regulation of bacterial infection, angiogenesis, and osteogenesis. ACS Appl Mater Interfaces. 2023;15:52276–89. 10.1021/acsami.3c11995 37920934 10.1021/acsami.3c11995

[27] Kokubo T. Bioactive glass ceramics: properties and applications. Biomaterials. 1991;12:155–63. 10.1016/0142-9612(91)90194-F 1878450 10.1016/0142-9612(91)90194-f

[28] Yu D, Guo S, Yang D, Li B, Guo Z, Han Y. Interrod spacing dependent angiogenesis and osseointegration of Na_2_TiO_3_ nanorods-patterned arrays via immunoregulation. Chem Eng J. 2021;426:131187 10.1016/j.cej.2021.131187

[29] Feng T, Yam FK. The influence of hydrothermal treatment on TiO_2_ nanostructure films transformed from titanates and their photoelectrochemical water splitting properties. Surf Interfaces. 2023;38:102767 10.1016/j.surfin.2023.102767

[30] Xu Z, Li M, Li X, Liu X, Ma F, Wu S, et al. Antibacterial activity of silver doped titanate nanowires on Ti implants. ACS Appl Mater Interfaces 2016;8:16584–94. 10.1021/acsami.6b04161 27336202 10.1021/acsami.6b04161

[31] Wadge MD, Stuart BW, Thomas KG, Grant DM. Generation and characterisation of gallium titanate surfaces through hydrothermal ion-exchange processes. Mater Des. 2018;155:264–77. 10.1016/j.matdes.2018.05.060

[32] Zhou J, Wang X, Zhao L. Antibacterial, angiogenic, and osteogenic activities of Ca, P, Co, F, and Sr compound doped titania coatings with different Sr content. Sci Rep. 2019;9:14203 10.1038/s41598-019-50496-3 31578429 10.1038/s41598-019-50496-3 PMC6775141

[33] Al-Hammadi AH, Al-Adhreai AA, Abdulwahab AM, Al-Adhreai A, Salem A, Alaizeri ZM, et al. An investigation on the structural, morphological, optical, and antibacterial activity of Sr: CuS nanostructures. Sci Rep. 2024;14:25169 10.1038/s41598-024-73701-4 39448649 10.1038/s41598-024-73701-4 PMC11502907

[34] Li C-Q, Yi S-S, Chen D-L, Liu Y, Li Y-J, Lu S-Y, et al. Oxygen vacancy engineered SrTiO_3_ nanofibers for enhanced photocatalytic H_2_ production. J Mater Chem A. 2019;7:17947–80. 10.1039/C9TA03701B

[35] Yuan Z, Guo M, Shi Q, Liang S, Chen Z, Wang S, et al. Preparation and piezoelectric assisted photocatalytic degradation of BaTiO_3_/SrTiO_3_ nanocomposites. Ceram Int. 2024;50:34890–34900. 10.1016/j.ceramint.2024.06.299

[36] Yin L, Zhou J, Gao L, Zhao C, Chen J, Lu X, et al. Characterization and osteogenic activity of SrTiO_3_/TiO_2_ nanotube heterostructures on microporous titanium. Surf Coat Technol. 2017;330:121–30. 10.1016/j.surfcoat.2017.09.075

[37] Gallab M, Le PHM, Shintani SA, Takadama H, Ito M, Kitagaki H, et al. Mechanical, bioactive, and long-lasting antibacterial properties of a Ti scaffold with gradient pores releasing iodine ions. Biomater Adv. 2024;158:213781 10.1016/j.bioadv.2024.213781 38335763 10.1016/j.bioadv.2024.213781

[38] Xu Z, Lu H, Lu J, Lv C, Zhao X, Wang G. Zhang, Enhanced osteogenic activity of Ti alloy implants by modulating strontium configuration in their surface oxide layers. RSC Adv. 2018;8:3051–60. 10.1039/c7ra10807a 35541194 10.1039/c7ra10807a PMC9077531

[39] Otitoju TA, Ahmad AL, Ooi BS. Superhydrophilic (superwetting) surfaces: a review on fabrication and application. J Ind Eng Chem. 2017;47:19–40. 10.1016/j.jiec.2016.12.016

[40] Park W, Müller S, Baumann RP, Becker S, Hwang B. Surface energy characterization of nanoscale metal using quantitative nanomechanical characterization of atomic force microscopy. Appl Surf Sci. 2020;507:145041 10.1016/j.apsusc.2019.145041

[41] Qi G, Liu X, Li C, Wang C, Yuan Z. The origin of superhydrophobicity for intrinsically hydrophilic metal oxides: a preferential O_2_ adsorption dominated by oxygen vacancies. Angew Chem Int Ed 2019;58:17406–11. 10.1002/anie.201909121 10.1002/anie.201909121 31556200

[42] Sahoo S, Sinha A, Balla VK, Das M. Synthesis, characterization, and bioactivity of SrTiO_3_-incorporated titanium coating. J Mater Res. 2018;33:2087–95. https://link.springer.com/article/10.1557/jmr.2018.99

[43] Knapic D, Minenkov A, Luczak W, Zrinski I, Kleber C, Hild S, et al. Hindrance of osteoblast cell adhesion on titanium by surface nanostructuring. Surf Interfaces. 2024;46:103990 10.1016/j.surfin.2024.103990

[44] Jiang S, Li W, Liu J, Jiang J, Zhang Z, Shang W, et al. ZnO@ZIF-8 core-shell structure nanorods superhydrophobic coating on magnesium alloy with corrosion resistance and self-cleaning. J Magnes Alloy. 2023;11:3287–301. 10.1016/j.jma.2022.01.014

[45] Ciobanu G, Harja M. Cerium-doped hydroxyapatite/collagen coatings on titanium for bone implants. Ceram Int. 2019;45:2852–7. 10.1016/j.ceramint.2018.07.290

[46] Zhu H, Guo D, Sun L, Li H, Hanaor DAH, Schmidt F, et al. Nanostructural insights into the dissolution behavior of Sr-doped hydroxyapatite. J Eur Ceram Soc. 2018;38:5554–62. 10.1016/j.jeurceramsoc.2018.07.056

[47] Lin Q, Huang D, Du J, Wei Y, Hu Y, Lian X, et al. Nano-hydroxyapatite crystal formation based on calcified TiO_2_ nanotube arrays. Appl Surf Sci. 2019;478:237–46. 10.1016/j.apsusc.2019.01.226

[48] Nie J, Zhou J, Huang X, Wang L, Liu G, Cheng J. Effect of TiO_2_ doping on densification and mechanical properties of hydroxyapatite by microwave sintering. Ceram Int. 2019;45:13647–55. 10.1016/j.ceramint.2019.04.007

[49] Bakhshi H, Sarraf-Mamoory R, Yourdkhani A, AbdelNabi AA, Mozharivskyj Y. Sol-gel synthesis, spark plasma sintering, structural characterization, and thermal conductivity measurement of heavily Nb-doped SrTiO_3_/TiO_2_ nanocomposites. Ceram Int. 2020;46:3224–35. 10.1016/j.ceramint.2019.10.027

[50] Brzezińska-Miecznik J, Jeleń P, Haberko K, Mozgawa W, Sitarz M. The effect of NaOH and KOH treatment on the behavior of CO_3_^2-^ and OH^-^ groups in natural origin hydroxyapatite. Ceram Int. 2017;43:12540–5. 10.1016/j.ceramint.2017.06.127

[51] Ionita D, Bajenaru-Georgescu D, Totea G, Mazare A, Schmuki P, Demetrescu I. Activity of vancomycin release from bioinspired coatings of hydroxyapatite or TiO2 nanotubes. Int J Pharmaceutics. 2017;517:296–302. 10.1016/j.ijpharm.2016.11.062 10.1016/j.ijpharm.2016.11.062 27913240

[52] Wang Y, Teng W, Zhang Z, Zhou X, Ye Y, Lin P, et al. A trilogy antimicrobial strategy for multiple infections of orthopedic implants throughout their life cycle. Bioact Mater. 2021;6:1853–66. 10.1016/j.bioactmat.2020.11.030 33336116 10.1016/j.bioactmat.2020.11.030 PMC7732879

[53] Brauer DS, Karpukhina N, Kedia G, Bhat A, Law RV, Radecka I, Hill RG. Bactericidal strontium-releasing injectable bone cements based on bioactive glasses. J R Soc Interface. 6 (2013). 10.1098/rsif.2012.0647. 10.1098/rsif.2012.0647 PMC3565794 23097502

[54] Yi Q, Liang P, Liang D, Shi J, Sha S, Chang Q. Multifunction Sr doped microporous coating on pure magnesium of antibacterial, osteogenic and angiogenic activities. Ceram Int. 2021;47:8133–41.

[55] Wang H, Zheng T, Yang N, Li Y, Sun H, Dong W, et al. Osteogenic and long-term antibacterial properties of Sr/Ag-containing TiO_2_ microporous coating in vitro and in vivo. J Mater Chem B 2023;11:2972–88. 10.1039/D2TB01658C 36919628 10.1039/d2tb01658c

[56] Godoy-Gallardo M, Eckhard U, Delgado LM, de Roo Puente YJD, Hoyos-Nogués M, Gil FJ, Perez RA. Antibacterial approaches in tissue engineering using metal ions and nanoparticles: from mechanisms to applications. Bioact Mater. 2021;6:4470–90. 34027235 10.1016/j.bioactmat.2021.04.033 PMC8131399

